# Properties of Ferrite Garnet (Bi, Lu, Y)_3_(Fe, Ga)_5_O_12_ Thin Film Materials Prepared by RF Magnetron Sputtering

**DOI:** 10.3390/nano8050355

**Published:** 2018-05-22

**Authors:** Mohammad Nur-E-Alam, Mikhail Vasiliev, Vladimir Belotelov, Kamal Alameh

**Affiliations:** 1 Electron Science Research Institute, Edith Cowan University, 270 Joondalup Dr, WA 6027, Australia; m.vasiliev@ecu.edu.au (M.V.); k.alameh@ecu.edu.au (K.A.); 2 Faculty of Physics, Lomonosov Moscow State University, Leninskie Gory, Moscow 119991, Russia; v.i.belotelov@ya.ru

**Keywords:** magneto-optics, magnetic thin films, optical constants

## Abstract

This work is devoted to physical vapor deposition synthesis, and characterisation of bismuth and lutetium-substituted ferrite-garnet thin-film materials for magneto-optic (MO) applications. The properties of garnet thin films sputtered using a target of nominal composition type Bi_0.9_Lu_1.85_Y_0.25_Fe_4.0_Ga_1_O_12_ are studied. By measuring the optical transmission spectra at room temperature, the optical constants and the accurate film thicknesses can be evaluated using Swanepoel’s envelope method. The refractive index data are found to be matching very closely to these derived from Cauchy’s dispersion formula for the entire spectral range between 300 and 2500 nm. The optical absorption coefficient and the extinction coefficient data are studied for both the as-deposited and annealed garnet thin-film samples. A new approach is applied to accurately derive the optical constants data simultaneously with the physical layer thickness, using a combination approach employing custom-built spectrum-fitting software in conjunction with Swanepoel’s envelope method. MO properties, such as specific Faraday rotation, MO figure of merit and MO swing factor are also investigated for several annealed garnet-phase films.

## 1. Introduction

Yttrium Iron Garnet (YIG) is one of the most common and well-known iron garnet materials possessing unique functional properties suitable for magneto-optic and microwave-range radio frequency (RF) applications. It is chemically formulated as Y_3_[Fe_2_](Fe_3_)O_12_ where Y^3+^ ions occupy the dodecahedral sublattice sites, two of the Fe^3+^ ions reside in the octahedral sites, and the remaining three Fe^3+^ ions are in tetrahedral sublattice sites. Research efforts, focusing on the refinement of structure and composition of garnets through the addition of a number of elements into the garnet material system, have resulted in scientific and technological benefits for various emerging applications [1,2,3,4,5,6,7,8,9,10,11,12]. Many reports have demonstrated the successful synthesis of new garnet material types by substituting the yttrium with either the transition-metal or rare-earth ions, such as Bi^3+^, Ce^3+^, Er^3+^, Tb^3+^ and others, into the dodecahedral sites, and also replacing the Fe^3+^ ions by other elements (such as Ga^3+^, Al^3+^, or other metals) into the tetrahedral sublattice sites [13,14,15,16,17,18,19,20,21,22]. Also, several composite-type material systems have been explored to improve the optical and magneto-optical properties of highly bismuth-substituted ferrite garnet thin-film materials [23,24,25,26,27,28,29]. Bismuth substitution into the YIG-based garnet lattice structure enhances the Faraday rotation performance, while other added components like Ga or Al contribute to the preferential dilution of iron inside the octahedral and tetrahedral sites, which then reduces the net magnetization. Modified ferrite garnets, especially Bi-substituted iron garnets (with compositions close to Bi_3_Fe_5_O_12_) have very high specific Faraday rotations across the visible light spectrum and relatively low optical absorption in the near-infrared spectral region. Multiple process parameters relevant to the synthesis of substituted iron garnet materials have a significant influence on their physical (structural, optical and magnetic) properties [30,31,32,33]. However, it is always challenging to develop application-specific substituted ferrite garnet thin-film materials with high quality (in terms of their structural, optical and magneto-optical properties being optimized simultaneously) by using physical vapor deposition techniques. It is often difficult to keep the composition-dependent lattice parameter as close as possible to that of YIG (12.38 Å), and most of the magneto-optical devices require the lattice mismatch between the garnet layer and the substrates be very small, to ensure film properties and morphology being close to these obtainable with liquid-phase epitaxy techniques. Also, the accurate determination of complex refractive index (optical constants data) of each particular thin-film garnet material composition is crucial for the design and optimization of all MO and magnetic photonic crystal (MPC) based devices.

In this work, we report on the successful synthesis of bismuth and lutetium co-substituted ferrite garnet thin-film material using RF magnetron sputtering process followed by high-temperature annealing in air. Co-substituted (Bi, Lu) yttrium gallium-iron garnet films prepared using an oxide-mix-based ceramic sputtering target of stoichiometry type Bi_0.9_Lu_1.85_Y_0.25_Fe_4_Ga_1_O_12_ are investigated. The structural, optical, and magneto-optical properties of this material are investigated. Special attention is devoted to the determination and investigations of the optical constants (refractive index, absorption coefficient and extinction coefficient) of thin-film materials of this composition type across the visible and near-infrared spectral range. The reason for choosing this particular composition type of the sputtering target was to produce a garnet layer with its lattice parameter as close as possible to that of YIG, and also to obtain low coercivity for applications requiring magnetization-state switching such as MO imaging. The other motivation was to explore a new type of garnet material stoichiometry, particularly Bi_0.9_Lu_1.85_Y_0.25_Fe_4.0_Ga_1_O_12_, with a combined substitution of Bi and Lu ions at yttrium (Y) lattice sites, which has so far not been explored extensively using physical vapour deposition techniques.

## 2. Experimental Work

### 2.1. Thin Film Garnet Layer Preparation 

**Figure 1:**
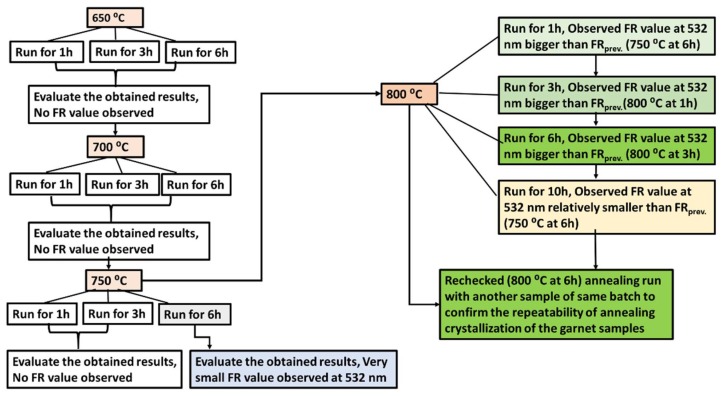
Schematic diagram of annealing process optimization experiments conducted to find the most suitable annealing regimes (in terms of both the maximum process temperature and crystallization process duration) for this type of garnet layers.

Several batches of ferrite garnet thin films were deposited on cleaned glass (1 mm thick) substrates from an oxide-base-mixed target of composition type Bi_0.9_Lu_1.85_Y_0.25_Fe_4.0_Ga_1_O_12_ (manufactured by Zhongnuo Advanced Material (Beijing, China) Technology Co., Ltd.). Each batch consisted of at least 4–6 small cleaned glass substrates (1.5 × 1.5 cm^2^) to deposit thin garnet layers. Specifically, the garnet thin films were sputtered using RF gun power of 250 W (5.4 W/cm^2^ at the surface of 7.62 cm (3 inch) diameter target) inside a high-vacuum chamber. The chamber pressure was kept at around 266.645 Pa (2 mTorr) with continuous flow (at 12 sccm) of pure argon (Ar). The substrates were kept at room temperature (21–23 °C), with a substrate stage rotation rate being about 15.5 rpm. The distance between the sputtering target and the substrates stage was kept at 18 cm. The deposition run time of each batch varied from 2 to 4 h. The growing film thicknesses of the layers were measured directly in-situ during the deposition using a quartz microbalance sensor, which was located very close to the substrates stage inside the sputtering chamber. The thickness sensor was calibrated using a series of preliminary deposition runs followed by optical characterization. Different annealing regimes were trialled using a conventional annealing oven at an ambient air atmosphere in order to find the optimized annealing regime (in terms of both the crystallization temperature and process duration) for this garnet composition. Finding the most suitable annealing regime for each newly-trialled garnet composition type is always a key factor for obtaining garnet thin films demonstrating a combination of maximized specific Faraday rotation and optical transparency. Figure 1 presents the schematic diagram of process sequence employed to identify the most suitable annealing crystallization process. The ramp rates of heating and cooling applied during the oven-annealing process were kept at a constant value of about 5 °C per minute in this work. We found that for this garnet composition type, the best optical and MO quality in films was obtained after running the annealing process for 6 h at 800 °C.

### 2.2. Structural Investigation

The crystal structure type and the crystallized garnet-phase lattice parameters of all annealed films were investigated using X-ray diffractometry (XRD). The experiments were performed by using a Siemens 5000D X-ray diffractometer at room temperature. This diffractometer was operated in theta-theta geometric configuration; the X-ray source was run at 40 kV and 30 mA. It generated a Cu Kα_1_ collimated X-ray beam that provided radiation at the wavelength λ = 0.154056 nm. Measurements were performed using a range of 2θ angles between 20° and 70°, at near-grazing X-ray radiation incidence, which allowed reliable measurements despite small sample thickness, and led to detecting multiple diffraction peaks coming from the samples, the signal strengths of which were sufficient for reliable indexing of the diffraction pattern. From the data obtained, the lattice constant and the averaged crystallite size were calculated by using the methods described in Refs [34,35]. Energy dispersive spectroscopy (EDS) based elemental analysis experiments were also performed, using a Quantax Q100 system (Bruker.com), to determine the elemental composition in the garnet films. The measurement system used for EDS experiments, was calibrated with a standard Cu sample provided by the manufacturer.

### 2.3. Optical Properties Measurement

The optical transmission spectra of both the as-deposited and annealed garnet films were measured and recorded using a UV/Visible spectrophotometer (Agilent Cary 5000). The transmission spectra measurements were carried out at room temperature at normal incidence for all the garnet samples in a wavelength range from 300 nm to 2500 nm. The obtained optical transmittance data were used to estimate the optical constants by using the well-known analysis method suggested by Swanepoel (established on the basis of the Manifacier idea [36]) which is suitable to calculate the optical constants of semi-transparent thin-film materials with relatively higher transparency (weak and medium absorptive media) [37,38,39]. The transmittance spectra envelopes were generated digitally to get the interference maxima (T_M_), interference minima (T_m_) to determine the refractive index (n) values as well as the film thickness. Using the calculated refractive index values (SWEM) in Cauchy’s dispersion equation the refractive index over the spectral range 300–2500 nm were determined. Later, the transmission spectra were modelled with help of MPC software [25,27] using the refractive index data (obtained from Cauchy’s formula) and re-fitted with experimentally obtained transmission spectra of the garnet films. From the iterative fitting of transmission spectra (modelled and experimental), the physical layer thicknesses and also the absorption coefficients of garnet films were derived.

### 2.4. Magneto-Optical Properties Measurement

The specific Faraday rotation values and magnetic hysteresis loop data for the annealed garnet layers were measured using a Thorlabs PAX polarimeter system in-conjunction with a custom-made calibrated electromagnet with an applied external magnetic field. The applied magnetic field direction during the measurement process was kept perpendicular to the film plane and parallel to the light propagation. The specific Faraday rotation and MO figure of merit of the annealed garnet films was calculated using the following expressions:

*Specific Faraday rotation* Θ_F_ = *Rotation angle (one way)*/*Film thickness* (°/μm) (1)

*MO figure of merit Q* = 2* Θ_F_/α (deg) (2)

where α is optical absorption coefficient at the same wavelength.

The MO figures of merit were calculated by taking into account the measurement errors in the films’ thickness (within estimated ±5% accuracy) as well as in Faraday rotation angles (measured with a maximum error of ±0.05°) and presented with the possible error bars.

## 3. Results

### 3.1. X-ray Diffraction Study

**Figure 2:**
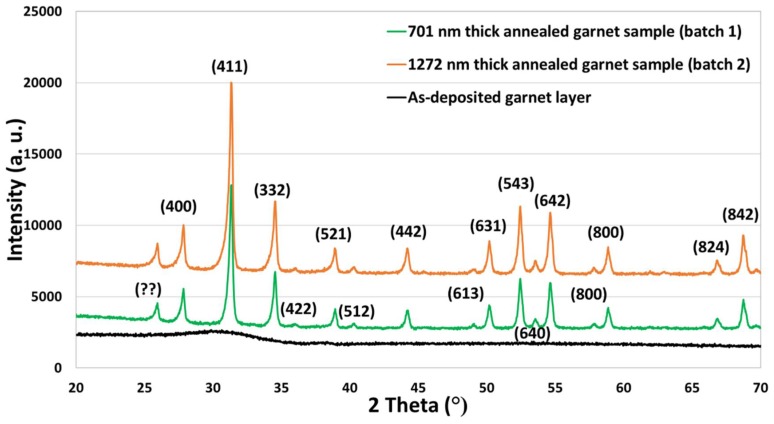
X-ray diffractograms of as-deposited and annealed garnet layers.

The X-ray diffractograms of as-deposited and annealed garnet thin layers are illustrated in Figure 2. It was observed that the as-deposited garnet layers did not have any identifiable peaks which confirms the amorphous phase of the samples just after deposition. On the other hand, high temperature annealed samples showed high-intensity diffraction peaks for wide range of diffraction angles. The positions of the X-ray diffraction peaks obtained from the annealed garnet (garnet-phase) samples were determined by using the software called “JADE 9” (MDI Corporation). The annealed garnet samples showed a nano-crystalline microstructure nature and the analysis of the XRD pattern revealed the presence of a crystal structure with a body-centered cubic (bcc) lattice type.

The experimentally measured lattice parameter (average 12.39 Å) of this garnet type material was found to be close to the predicted lattice parameter of a garnet layer of this composition type described by the stoichiometry Bi_0.9_Lu_1.85_Y_0.25_Fe_4_Ga_1_O_12_. Theoretically predicted crystal lattice parameter for this type of doped iron-garnet material was (𝐴_0_) = 12.376 (Å) + 0.0828 × 0.9 (Å) − 0.031 × 1.85 (Å) − 0.0151 × 1 (Å) = 12.378 (Å), where 12.376 (Å) is the lattice parameter of Y_3_Fe_5_O_12_ (YIG). The average crystallite size of the annealed garnet films was calculated using Scherrer equation, Dp = Kλ/βcosθ [34,35,40] where the value for the shape factor K is 0.94 and the X-ray wavelength λ = 0.154056 nm were used. The measured average crystallite size for this garnet film was 33.9 nm.

### 3.2. EDS Measurement

**Figure 3:**
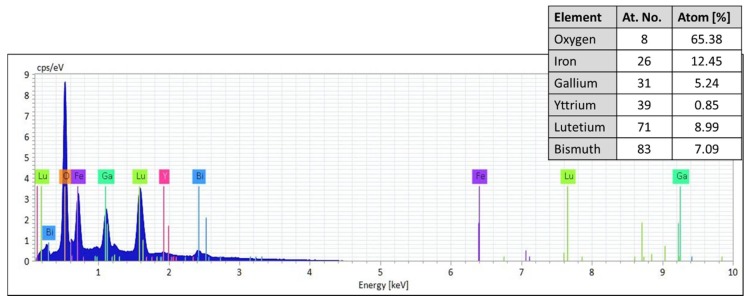
A typical energy dispersive X-ray (EDS) spectrum of a garnet (as-deposited) sample with the characteristics peaks for each possible element present in the film. The inset shows the obtained constituent elements (atomic %) in the sample. To avoid any type of contamination such as by carbon or other substances/gases, the measurement time was limited (at 5 min maximum) according to the manufacturer recommendations provided in the system manual. Cps: counts per second.

Figure 3 presents a typical energy dispersive spectroscopy (EDS) spectrum of a garnet (as-deposited) sample displaying the Y-axis representing the counts per second (number of X-rays received and processed by the detector) and X-axis presenting the energy level of those counts. EDS microanalysis typically helps to identify the particular elements belongs to the garnet samples and their relative proportions (in atomic %). EDS experiments were performed using a beam power of 10 keV. All peaks of the expected elements (Bi, Lu, Y, Fe, Ga and O) were seen very clearly on the measured spectra. These results confirm the introduction of both Bi and Lu ions into the garnet structure. Also, a large oxygen content (>60 at. %) was measured in all films which could be the cause of experimental errors in measuring the elemental content of other atoms, or extra oxygen could, in fact, be present either within the substrates or trapped (from air) within the film pores.

### 3.3. Study of Optical Properties

#### 3.3.1. Optical Transmittance

**Figure 4:**
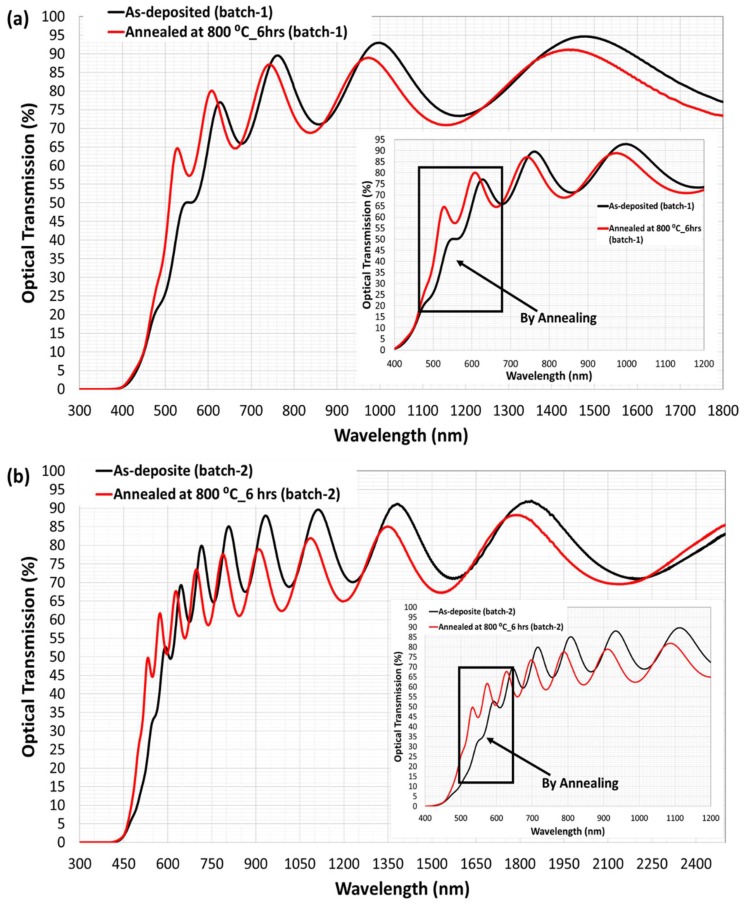
Measured transmission spectra of as-deposited and annealed garnet samples of about 701 nm (**a**), and (**b**) 1272 nm thickness. The insets of the figures showed the shift in the absorption edge to lower wavelengths due to the annealing crystallization process. The shift of the band gap due to the annealing process attributed to remove the residual stresses of the garnet layers and improved the structural and crystalline quality of the annealed garnet films.

The transmittance spectra of as-deposited and annealed garnet thin films of two different thicknesses (indicated in the figure caption) are shown in Figure 4a,b. Clear and distinct interference fringes are observed in the transparent region (longer wavelengths section) with a relatively large intensity above 90%. The intensity of the transmission of the interference fringes starts to decrease in amplitude moving towards the shorter wavelengths due to the beginning of absorption within the films and continues towards zero at the fundamental absorption edge of the film. The number of the observed interference fringes was found to be about 4–5 fringes only for the relatively thin samples (701 nm) and 6–7 for the samples with higher thickness (1272 nm). The number of interference fringes in the transmission spectrum were found to be fully dependent on the film thickness as reported in Ref [41]. The appearance of adequate interference patterns in the measured transmittance spectra of the samples (as-prepared and annealed) also revealed that high quality garnet thin films were obtained. Figure 4 also revealed that although transmittance became slightly reduced in the longer wavelength range due to the annealing process, it improved the transmittance significantly across the short wavelength range (at around 450–620 nm), which indicated directly the lower absorption in the crystallized garnet samples.

#### 3.3.2. Calculation of the Refractive Index and Film Thickness

**Figure 5:**
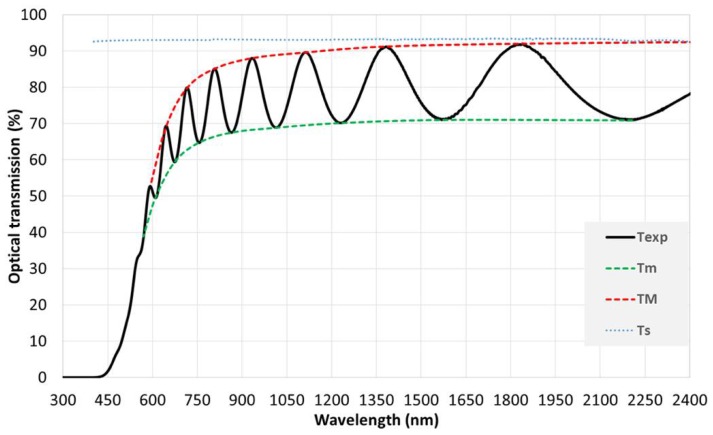
Typical transmission spectrum for the as-deposited garnet sample; where T_exp_ is the measured transmission of the sample, T_M_ and T_m_ are the maxima and minima of the envelopes. Transmission spectrum of 1 mm thick glass substrate is also included in the figure.

The spectral dependence of the refractive index (n) garnet samples was calculated using Swanepoel’s envelope method (SWEM). The theory of SWEM are extensively described in Refs [36,37,38,39]. Figure 5 presents the typical transmission spectrum of an as-deposited garnet film including the envelopes transmission maxima (T_M_) and minima (T_m_) intensities.

To calculate the approximate values of refractive index (n_1_) of garnet films and for the clear glass substrate the following well-known basic set of expressions were used:

*n*_1_ = *[N + (N*^2^ − *S*^2^*)*^1/2^*]*^1/2^ (3)

*N* = *2S{(T_M_ − T_m_)/T_M_·T_m_} + (S*^2^ + *1)/2* (4)

where T_M_ and T_m_ denote the maximum and minimum transmittance at a given wavelength, respectively. Parameter S represents the refractive index of used glass substrate. The necessary values of the refractive index of the glass substrate (S) were obtained from the measured transmission spectrum using the following equation:

*S* = *1/T_S_ + (1/T_S_*^2^*− 1)*^1/2^ (5)

Here T_S_ represents the transmission coefficient of the substrate. No significant dispersion was present in the transmission spectrum of the transparent glass substrate (Figure 5). The average calculated n value for the used glass substrate was around 1.47. Using Equation (3), the primary refractive index (n_1_) values were calculated as shown in Table 1 (column 5). The order number of interference fringes was determined by substituting n_1_ values into the basic equation of the interference fringes:

*2nd* = *m*_0_λ (6)

where m_0_ is called the order number and it is equal to an integer for maxima and a half-integer for minima. By using the wavelength and refractive index values of wavelengths corresponding to two adjacent maxima (or minima), the first approximation value of the film thickness (d_1_) were calculated (Table 1 column 6) using the following expression:

*d_l_* = λ_1_λ_2_/*2*(*n*_2_λ_1_ − *n*_1_λ_2_) (7)

After calculating the first approximated values of the refractive index and film thickness, we followed the evaluation method as described in Ref [37,38] to reduce the bigger deviation obtained in first-approximation film thickness. The final film thickness (d_2_ in Table 1, column 9) was found with a smaller dispersion (less than 1%), and this was in agreement with the literature and helped to determine the final refractive index values (n_2_ listed in Table 1, column 10) with better accuracy. Below, Table 1 summarizes the calculated optical parameters for the first batch (as-deposited and annealed) garnet thin films.

It can be noted in Table 1 that the calculated film thickness is larger than that measured by the quartz sensor. Later, we recalculated the tooling factor (TF), which is one of the important deposition process-control parameters, by using the correct film thickness (701 nm as obtained from the best transmission-spectrum fit obtained over the entire spectral range of measurement) and used the newly calculated TF for the second batch of garnet samples. We deposited a garnet layer of thickness around 1292 nm onto glass substrates and followed the same SWEM to determine the optical constants and the film thickness of the sample. The results obtained from the batch-2 samples are detailed in Table 2.

**Figure 6:**
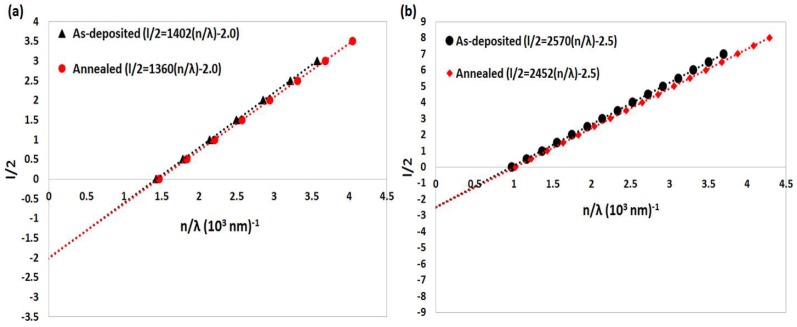
Plots of (l/2) versus (n/λ) to determine the physical film thickness and the first-order value m_1_ for as-deposited and annealed garnet thin films, according to the modified Equation (8). Film thickness (**a**) 701 nm, and (**b**) 1272 nm.

The calculated film thickness (1285 nm, batch 2 sample) was now found to be much closer to that measured by the quartz sensor (1292 nm) during the deposition process. With the thicker films, a slightly higher variation in the film thicknesses obtained by the two techniques was observed (about 2%), compared to that of the thinner films (701 nm, batch 1). This may be due to the formation of some non-uniformities within the film layers, or due to larger pores present within thicker films formed during the deposition process. However, after examination of the calculated n and d values for all samples, and according to data reported in [37,38], it was found that the following expression (Equation (8)) is more accurate than Equation (6) for the evaluation of interference fringes:

*l/2* = *2d (n/*λ*) − m*_1_ (8)

where m_1_ is the first order value, which equals an integer for a maximum and a half integer for a minimum, and l = 0, 1, 2, 3, …. Figure 6a,b show plots of (l/2) versus (n/λ) which are used to determine the physical film thickness and the first-order value m_1_, for as-deposited and annealed garnet thin films, according to the modified Equation (8). The plots shown in Figure 6a,b validate Equation (8), and enable the calculation of the film thickness d (which is half of slope value) and the first order m_1_ value.

**Figure 7:**
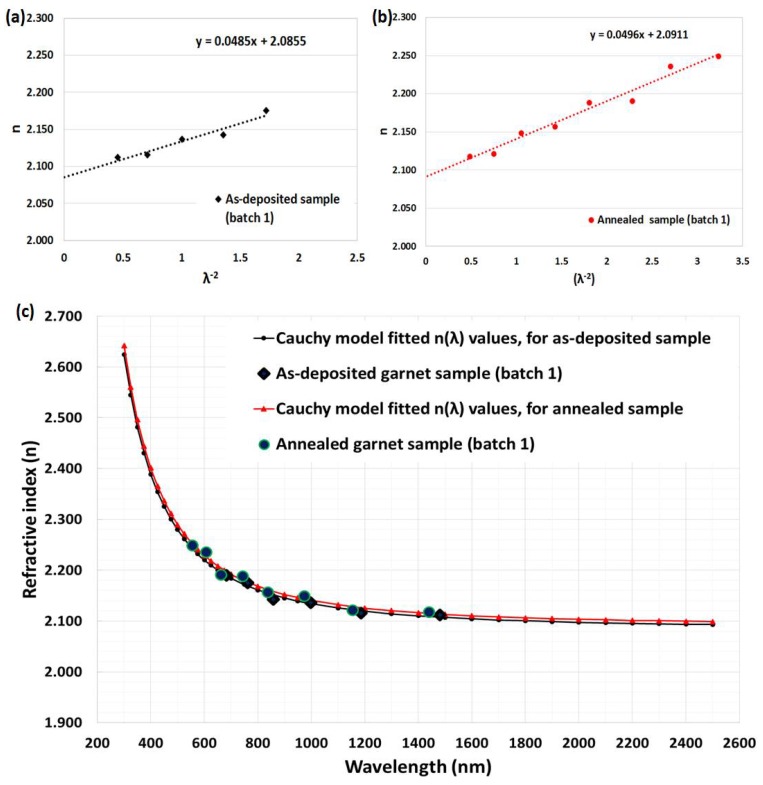
Least square fit of calculated refractive index (n_2_) values for garnet samples (batch-1); (**a**) as-deposited and (**b**) annealed. (**c**) Calculated refractive index spectra using Cauchy’s model and measured transmission spectra, for as-deposited and annealed samples.

**Figure 8:**
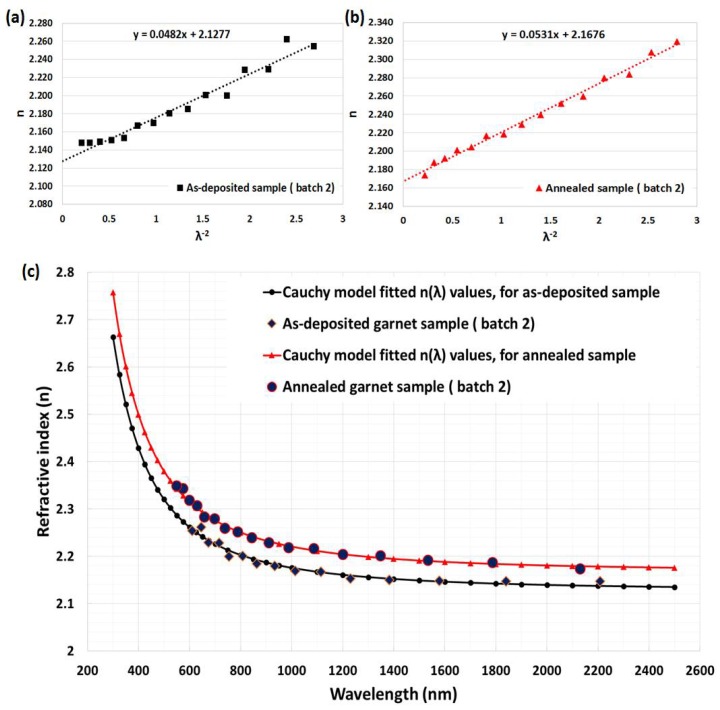
Least square fit of calculated refractive index (n_2_) values for garnet samples (batch-2); (**a**) as-deposited and (**b**) annealed. (**c**) Calculated refractive index spectra using Cauchy’s model and measured transmission spectra, for as-deposited and annealed samples.

Note that if the Cauchy’s constants are known, then the Cauchy’s dispersion formula can be used to determine the values of refractive index over the spectral range of 300–2500 nm [42,43]. By using the calculated values of the refractive index (n_2_) listed in Table 1 and Table 2, in conjunction with least-square fitting, according to Cauchy’s dispersion equation, the simple two-term expression was found to be accurate for the calculation of the refractive index:

*n (*λ*) = a + b/*λ^2^ (9)

where a and b are Cauchy’s constants, which can be determined from the intercept and slope of the n-versus-λ^−2^ linear plot, respectively. Figure 7a,b and Figure 8a,b show plots of n vs λ^−2^ for as deposited and annealed garnet samples from both batches. From these linear plots, the values of Cauchy’s constants (a and b) can be determined easily. By substituting the a and b values into Equation (9), the values of the refractive index can be derived over the whole spectral range of measurement, 300–2500 nm, for all garnet samples, as shown in Figure 7c and Figure 8c. In Figure 7c and Figure 8c, the n_2_ values are represented as blue solid large points, while n values, which were calculated from Cauchy’s dispersion relation, are shown as solid lines.

**Figure 9:**
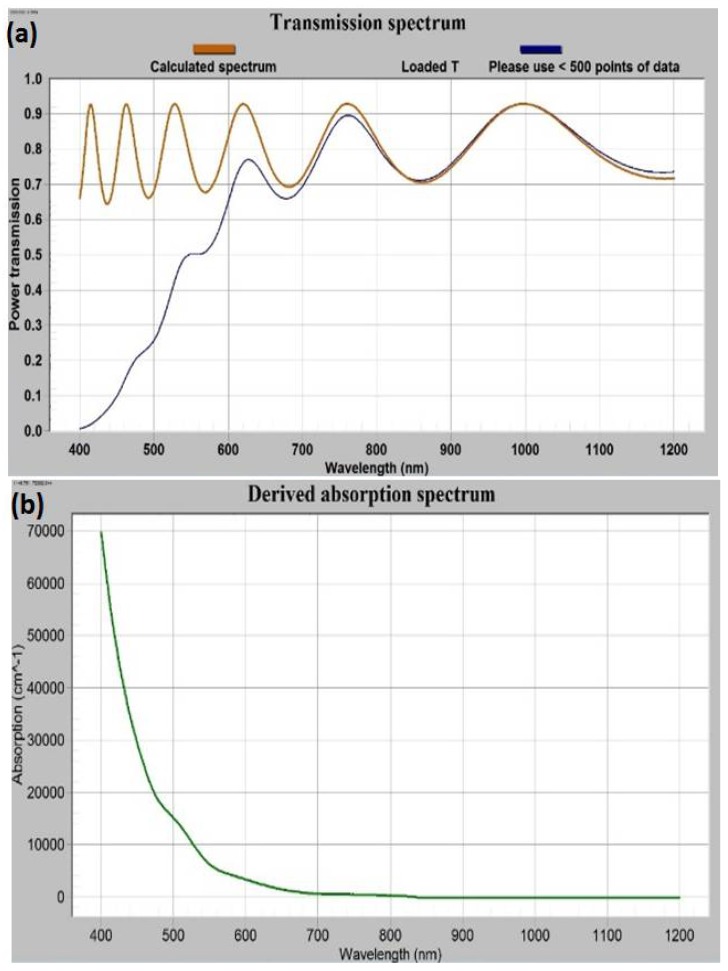
Iterative software-assisted fitting of modelled and measured transmission spectra (**a**), and (**b**) the derived absorption coefficient (α) of an as-deposited garnet sample grown on glass substrate from batch-1.

The impact of thermal annealing on the refractive index was obvious from Figure 7c and Figure 8c. We used the refractive index data (derived according to Cauchy’s formula for as-deposited sample batch-1) in our in-house built magneto-photonic crystal (MPC) software, which was reported in [25,27], to model and simultaneously fit the transmission spectrum of as-deposited sample, as shown in Figure 9a. From the iterative fitting of transmission spectra (by way of adjusting the physical thickness of modeled film and considering the absorption coefficient value equal to zero) we derived the best-fitted film thickness which was 701 nm (identical to that found in the SWEM calculation Table 1). Later the best-fitted absorption coefficients, shown in Figure 9b, were derived with the help of the MPC software (across the spectral range 300–1200 nm) by using the measured transmission spectrum data of the sample.

#### 3.3.3. Confirmation of the Film Thickness, Optical Absorption Coefficient Data and Extinction Coefficient Measurement

**Figure 10:**
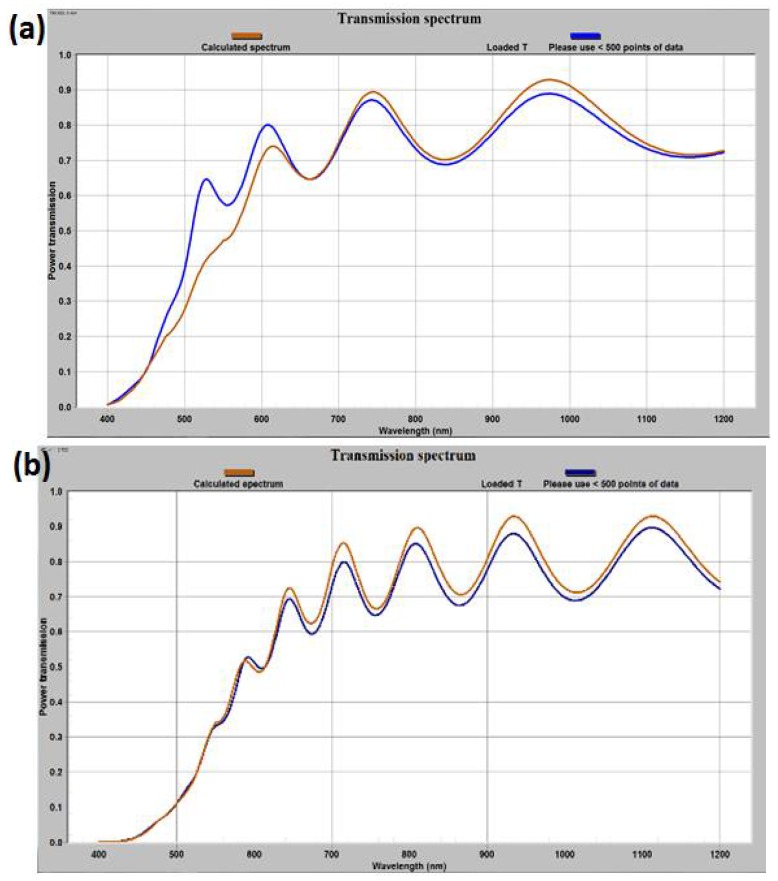

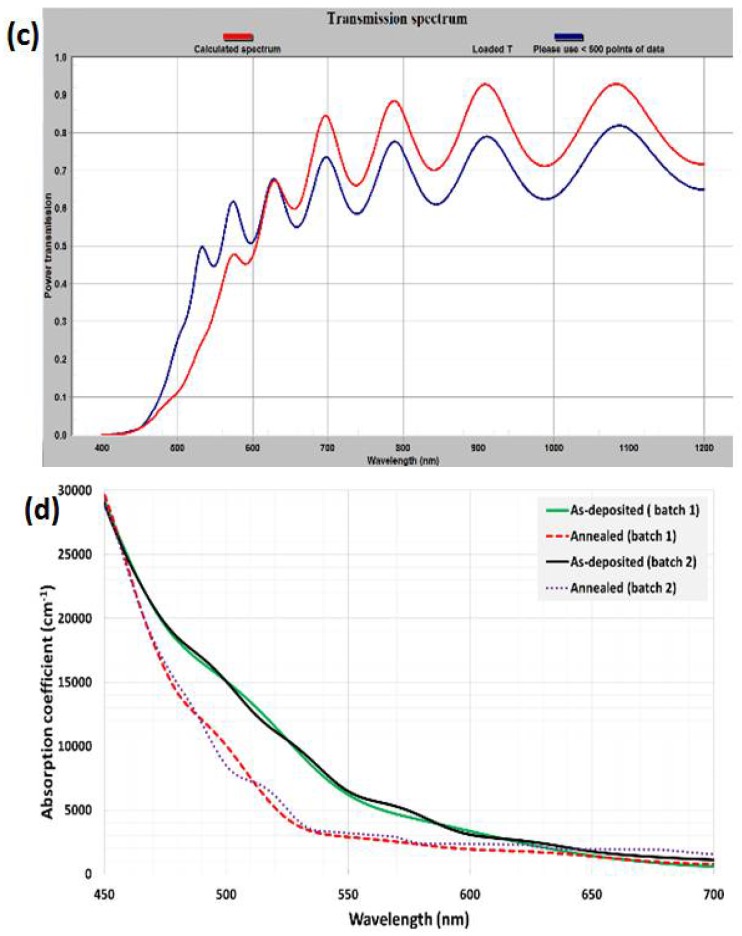
Magneto-photonic crystal (MPC) software fitted transmission spectra of different thin film garnet layers; (**a**) annealed garnet sample from batch-1, (**b**,**c**) as-deposited and annealed garnet samples form batch-2, (**d**) derived absorption coefficient datasets obtained for the as-deposited and annealed garnet samples.

We modelled and fitted the transmission spectra for other as-deposited and annealed garnet samples (from batches 1 and 2), using the refractive index data (derived according to Cauchy’s dispersion formula) together with the derived absorption coefficient (for as-deposited sample batch-1), with the help of MPC software. Figure 10a–c represents the best fitting results of transmission spectra with their respective modelled transmission spectra, which also re-confirmed our calculated refractive index and absorption coefficient datasets for this type of garnet thin films. From the fitting of the transmission spectra, the film thicknesses were recalculated (and accounted for later in measurements of specific Faraday rotation, MO figure of merit and swing factor of photoresponse) and the absorption coefficient data for other annealed and non-annealed samples were derived. Figure 10d shows the derived absorption coefficient data for all batches of sample (as-deposited and annealed). Figure 10d reveals that both annealed garnet films (batch 1 and 2) have similar patterns of optical absorption coefficient (α), which is in the range of 1630–3800 cm^−1^ over the visible spectral region.

**Figure 11:**
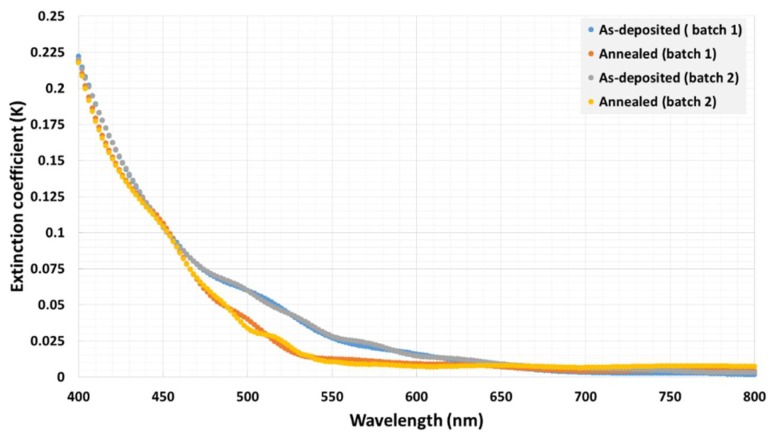
Spectral dependence of the extinction coefficient for as-deposited and annealed garnet samples (batch 1 and 2).

In addition, the extinction coefficient or the absorption index (k) data were investigated and found to follow similar trends as the absorption coefficient of the garnet samples (decreasing in k values with increasing the wavelength), thus confirming the low optical losses at longer wavelengths. The extinction coefficient data for these garnet samples were calculated using the simple formula k = αλ/4π, where α is the derived absorption coefficient (Figure 11). Note that the extinction coefficient is an important parameter in optical and magneto-optical thin-film materials related studies, such as electromagnetic wave propagation through structured solid thin-film media, because it controls the decay in the oscillation amplitude of the incident electric field.

### 3.4. Study of Magneto-Optical Properties

#### 3.4.1. Specific Faraday Rotation and MO Figure of Merit

**Figure 12:**
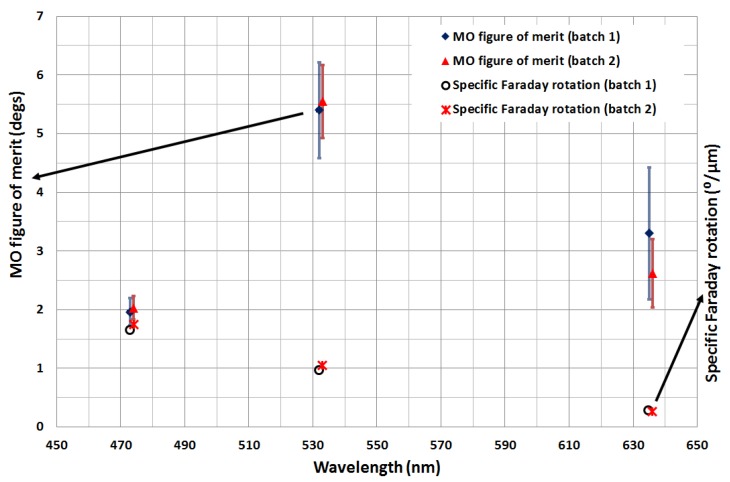
Measured specific Faraday rotation data and magneto-optic (MO) figures of merit at 473 nm, 532 and 635 nm.

The specific Faraday rotation is one of the key parameters that is used to assess the quality of the annealed garnet thin-film materials. The specific Faraday rotation performance was measured at the visible wavelengths 473 nm, 532 nm and 635 nm. All of the optimally annealed garnet films (batches 1 and 2) exhibited relatively high specific Faraday rotations as well as relatively low absorption coefficients. The best measured specific Faraday rotation and MO figure of merit values obtained at the above-mentioned wavelengths are plotted in Figure 12.

#### 3.4.2. MO Swing Factor and Hysteresis Loop of Specific Faraday Rotation

**Figure 13:**
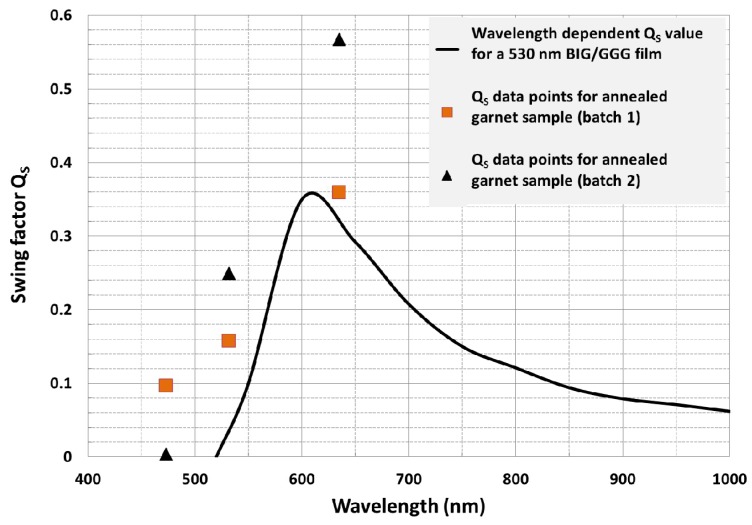
Measured swing factor of photo-response (Q_S_) data points at 473 nm, 532 and 635 nm are presented against the Q_S_ values of a 530 nm BIG/GGG film. The Q_S_ values for the Bismuth iron garnet film (Bi_3_Fe_5_O_12_) on Gadolinium Gallium garnet substrate ((Gd_3_Gd_5_O_12_) (BIG/GGG) film are reproduced digitally from the published result in Ref. [44].

**Figure 14:**
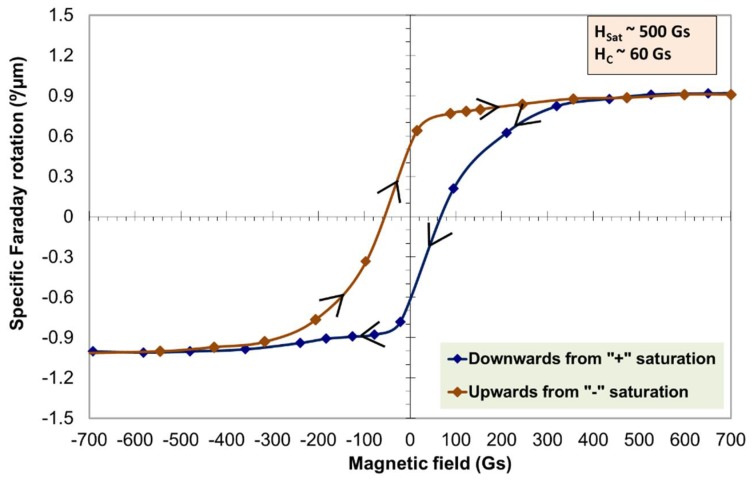
Measured hysteresis loop of Faraday rotation at 532 nm of a 1.272 µm thick annealed garnet sample.

The optical efficiency of MO visualization, which is also known as the swing factor of the photoresponse, was calculated using the measured specific Faraday rotation and the absorption coefficient. Figure 13 shows the measured swing factor (QS) of the photo-response at 473 nm, 532 and 635 nm and that of a 530 nm Bismuth iron garnet film (Bi_3_Fe_5_O_12_) on Gadolinium Gallium garnet substrate ((Gd_3_Gd_5_O_12_) (BIG/GGG) film. The Swing factor is defined as the ratio of change in optical signal intensity (induced by propagation through the MO medium) to the intensity of the incident polarized light, and can be calculated using the following expression Qs = abs[exp(−2αd)sin^2^(4ϴ_F_d)], where α is the absorption coefficient, ϴ_F_ is the specific rotation and d is film thickness [44]. Higher Qs factor values were observed for the sputtered garnet films compared to the values attained with a 530 nm BIG/GGG film (as shown in Figure 13). Figure 14 shows the measured hysteresis loop of Faraday rotation at 532 nm of an annealed garnet sample of thickness 1.272 µm. Note that, although the Qs factor is dependent on the film thickness, in contrast to the MO figure of merit, the high swing factor attained by our developed garnet films, together with the low coercive force (magnetic field) of <100 Gs (0.01 T) (as shown in Figure 14), makes this type of material attractive for the development of magneto-optical visualization devices.

Note also that, as shown in Figure 14, the measured coercive force and the saturation magnetization values were 60 Gs and 500 Gs, respectively. The almost square shape of the hysteresis loop and the strong remanence property indicate that the magnetization of this garnet material is perpendicular (out-of-plane).

## 4. Conclusions

We have successfully fabricated garnet thin-films sputtered using a target of nominal stoichiometry Bi_0.9_Lu_1.85_Y_0.25_Fe_4.0_Ga_1_O_12_ and crystallized using high-temperature oven-annealing processes. The optical properties, mainly the spectral dependency of the refractive index for this type of garnet material, have been calculated using only the transmission spectra. The methods used for the derivation of optical constants data have been described. All of the annealed garnet films have exhibited relatively high specific Faraday rotations, as well as relatively low absorption coefficients (i.e., high figures of merit). We have also investigated the magnetic and MO properties of the developed garnet films. The high swing factor and the low coercive force of the developed garnet films make them attractive for numerous applications, including magneto-optical visualizers and integrated optics.

## Figures and Tables

**Table 1:** Optical parameters obtained from the optical transmission measurements carried out using the first batch (as-deposited and annealed) of garnet samples.

Sample	Wavelength (nm)	T_M_	T_m_	n_1_	d_1_ (nm)	m_0_	M	d_2_ (nm)	n_2_
**As-deposited** (thickness **650 nm**, measured by a quartz sensor during deposition)	1480	0.94699	0.735	2.11	-	2.0	2.00	700	2.112
	1186	0.9465	0.73351	2.12	710	2.5	2.50	700	2.116
	998	0.92997	0.72	2.13	692	3.0	3.00	704	2.136
	858	0.928	0.71101	2.15	694	3.5	3.50	698	2.143
	762	0.89588	0.685	2.17	712	4.0	4.00	701	2.175
	682	0.86	0.65927	2.19	749	4.5	4.50	701	2.190
	628	0.77032	0.6	2.21	-	-	-	-	-
**d_1_ (ave) = 712 nm, δ_1_ = 22.8 nm (3.21%), d_2_ (ave) = 701 nm, δ_2_ = 1.8 nm (0.25%), d_(mpcmf)_ = 701 nm**									
**Annealed at 800 °C for 6 h**	1440	0.91145	0.71	2.124	-	2.0	2.00	678	2.118
	1154	0.91	0.70869	2.126	697	2.5	2.50	679	2.122
	974	0.8894	0.693	2.136	692	3.0	3.00	684	2.149
	838	0.888	0.68786	2.149	684	3.5	3.50	682	2.157
	744	0.87132	0.67	2.175	654	4.0	4.00	684	2.189
	662	0.845	0.64618	2.204	681	4.5	4.50	676	2.191
	608	0.80114	0.615	2.224	658	5.0	5.00	684	2.236
	556	0.75	0.57274	2.274	710	5.6	5.50	672	2.249
	528	0.64661	0.505	2.303	-	-	-	-	-
**d_1_ (ave) = 682 nm, δ_1_ = 17.7 nm (2.58%), d_2_ (ave) = 680 nm, δ_2_ = 4.3 nm (0.63%), d_(mpcmf)_ = 684 nm**									

**Table 2:** Optical parameters obtained from the optical transmission measurements carried out using the second batch (as-deposited and annealed) of garnet samples.

Sample	Wavelength (nm)	T_M_	T_m_	n_1_	d_1_ (nm)	m_0_	M	d_2_ (nm)	n_2_
**As-deposited** (thickness **1292 nm**, measured by a quartz sensor during deposition)	2208	0.905	0.70915	2.115	-	2.3	2.5	1305	2.148
	1840	0.92007	0.715	2.125	1264	2.8	3.0	1299	2.148
	1578	0.918	0.71022	2.136	1247	3.3	3.5	1293	2.149
	1382	0.91221	0.702	2.150	1251	3.8	4.0	1285	2.151
	1230	0.9122	0.7	2.156	1302	4.3	4.5	1283	2.153
	1114	0.89607	0.689	2.161	1320	4.7	5.0	1289	2.167
	1014	0.896	0.6887	2.162	1318	5.2	5.5	1290	2.170
	934	0.88012	0.678	2.166	1314	5.6	6.0	1294	2.180
	864	0.878	0.6753	2.171	1339	6.1	6.5	1294	2.185
	808	0.85129	0.658	2.176	1273	6.5	7.0	1300	2.201
	754	0.84	0.64701	2.191	1247	7.1	7.5	1291	2.202
	716	0.78892	0.61	2.215	1218	7.5	8.0	1293	2.229
	674	0.77	0.59379	2.235	1010	8.1	8.5	1282	2.229
	646	0.69356	0.53	2.318	899	8.7	9.0	1254	2.262
	610	0.648	0.49496	2.362	1007	9.4	9.5	1227	2.255
	592	0.52763	0.414	2.419	-	-	-	-	-
**d_1_ (ave) = 1215 nm, δ_1_ = 138.5 nm (11%), d_2_ (ave) = 1285 nm, δ_2_ = 19.9 nm (2%), d_(mpcmf)_ = 1310 nm**									
**Annealed at 800 °C for 6 h**	2130	0.89	0.69541	2.129	-	2.4	2.5	1251	2.174
	1786	0.8811	0.685	2.145	1213	2.9	3.0	1249	2.188
	1534	0.87	0.67214	2.166	1353	3.4	3.5	1240	2.192
	1348	0.85031	0.658	2.174	1193	3.8	4.0	1273	2.201
	1200	0.845	0.64917	2.193	1091	4.4	4.5	1229	2.205
	1086	0.81894	0.631	2.203	1192	4.9	5.0	1232	2.217
	988	0.815	0.62326	2.223	1154	5.4	5.5	1222	2.219
	910	0.8	0.61	2.240	1180	5.9	6.0	1218	2.229
	844	0.79	0.6	2.257	1219	6.4	6.5	1215	2.240
	788	0.77543	0.59	2.263	1244	6.9	7.0	1219	2.252
	738	0.77	0.58519	2.270	1253	7.4	7.5	1219	2.260
	698	0.73543	0.562	2.283	1203	7.9	8.0	1223	2.280
	658	0.72	0.54988	2.297	1217	8.4	8.5	1217	2.284
	628	0.67623	0.521	2.312	1217	8.9	9.0	1222	2.308
	598	0.66	0.50732	2.334	1134	9.4	9.5	1217	2.319
	574	0.61721	0.476	2.367	986	9.9	10.0	1213	2.344
	548	0.58	0.44593	2.416	1063	10.6	10.5	1191	2.349
	532	0.49716	0.392	2.444	-	-	-	-	-
**d_1_ (ave) = 1185 nm, δ_1_ = 84.7 nm (7%), d_2_ (ave) = 1226 nm, δ_2_ = 18.5 nm (2%), d_(mpcmf)_ = 1272 nm**									
